# Bis{2-hydroxy­imino-*N*′-[1-(2-pyrid­yl)ethyl­idene]propanohydrazidato}zinc(II) dihydrate

**DOI:** 10.1107/S1600536810003351

**Published:** 2010-01-30

**Authors:** Yurii S. Moroz, Kateryna O Znovjyak, Iryna O. Golenya, Svetlana V. Pavlova, Matti Haukka

**Affiliations:** a National Taras Shevchenko University, Department of Chemistry, Volodymyrska Str. 64, 01601 Kyiv, Ukraine; b Department of Chemistry, University of Joensuu, PO Box 111, 80101 Joensuu, Finland

## Abstract

The title compound, [Zn(C_10_H_11_N_4_O_2_)_2_]·2H_2_O, was prepared by the reaction between Zn(CH_3_COO)_2_·2H_2_O and 2-hydroxy­imino-*N*′-[1-(2-pyrid­yl)ethyl­idene]propano­hydrazide (Hpop). The central Zn^II^ atom has a distorted tetra­gonal-bipyramidal coordination geometry formed by two amide O atoms and four N atoms of two azomethine and two pyridine groups. In the crystal, complex mol­ecules form layers parallel to the crystallographic *b* direction. The layers are connected by  O—H⋯N and O—H⋯O hydrogen bonds involving the solvent water mol­ecules.

## Related literature

For zinc(II)-containing complexes with similiar ligands, see: Petrusenko *et al.* (1997 ▶); Comba *et al.* (2002 ▶); Kasuga *et al.* (2003 ▶). For the structural parameters of amide derivatives of 2-hydroxy­imino­propanoic acid, see: Onindo *et al.* (1995 ▶); Sliva *et al.* (1997*a* ▶,*b* ▶); Mokhir *et al.* (2002 ▶); Moroz *et al.* (2009*a* ▶,*b* ▶). For the preparation and characterization of 3*d*-metal complexes with 2-hydroxy­imino-*N*′-[1-(2-pyrid­yl)ethyl­idene]propanohydrazone, see: Moroz *et al.* (2008*a* ▶,*b* ▶).
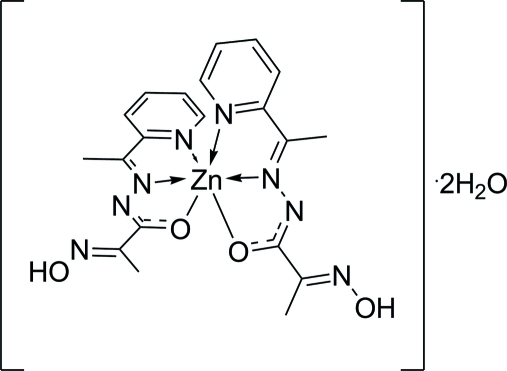


## Experimental

### 

#### Crystal data

[Zn(C_10_H_11_N_4_O_2_)_2_]·2H_2_O
*M* *_r_* = 539.86
Triclinic, 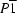

*a* = 8.3241 (3) Å
*b* = 10.6299 (4) Å
*c* = 13.9006 (5) Å
α = 94.184 (2)°
β = 101.389 (2)°
γ = 108.052 (2)°
*V* = 1134.48 (7) Å^3^
*Z* = 2
Mo *K*α radiation
μ = 1.14 mm^−1^
*T* = 100 K
0.28 × 0.07 × 0.02 mm

#### Data collection

Nonius KappaCCD diffractometer
Absorption correction: multi-scan (*SADABS*; Sheldrick, 2008 ▶) *T*_min_ = 0.743, *T*_max_ = 0.977
21551 measured reflections
5171 independent reflections
4253 reflections with *I* > 2σ(*I*)
*R*_int_ = 0.048

#### Refinement

*R*[*F* ^2^ > 2σ(*F* ^2^)] = 0.036
*wR*(*F* ^2^) = 0.092
*S* = 1.05
5171 reflections
320 parameters
H-atom parameters constrained
Δρ_max_ = 0.95 e Å^−3^
Δρ_min_ = −0.39 e Å^−3^

### 

Data collection: *COLLECT* (Bruker, 2004 ▶); cell refinement: *DENZO*/*SCALEPACK* (Otwinowski & Minor, 1997 ▶); data reduction: *DENZO*/*SCALEPACK*; program(s) used to solve structure: *SHELXS97* (Sheldrick, 2008 ▶); program(s) used to refine structure: *SHELXL97* (Sheldrick, 2008 ▶); molecular graphics: *ORTEP-3 for Windows* (Farrugia, 1997 ▶); software used to prepare material for publication: *SHELXL97*.

## Supplementary Material

Crystal structure: contains datablocks I, global. DOI: 10.1107/S1600536810003351/jh2129sup1.cif

Structure factors: contains datablocks I. DOI: 10.1107/S1600536810003351/jh2129Isup2.hkl

Additional supplementary materials:  crystallographic information; 3D view; checkCIF report

## Figures and Tables

**Table:** 

Zn1—N2	2.061 (2)
Zn1—N6	2.085 (2)
Zn1—O1	2.0880 (15)
Zn1—O3	2.1470 (15)
Zn1—N5	2.1955 (19)
Zn1—N1	2.2877 (19)

**Table:** 

N2—Zn1—O1	76.10 (7)
N6—Zn1—O3	74.17 (6)
N6—Zn1—N5	75.07 (7)
N2—Zn1—N1	73.97 (7)

**Table 2:** Hydrogen-bond geometry (Å, °)

*D*—H⋯*A*	*D*—H	H⋯*A*	*D*⋯*A*	*D*—H⋯*A*
O2—H2*O*⋯N7^i^	0.92	1.89	2.801 (3)	170
O4—H4*O*⋯O5^ii^	0.93	1.77	2.675 (3)	164
O5—H5*P*⋯O3	0.91	1.93	2.811 (3)	161
O5—H5*O*⋯O6	0.86	2.08	2.889 (3)	157
O6—H6*O*⋯N4^iii^	0.92	2.12	2.934 (3)	148
O6—H6*P*⋯N8^ii^	0.93	2.10	2.971 (3)	154

Symmetry codes: (i) 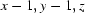; (ii) 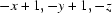; (iii) 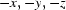.

**Fig. 1.**
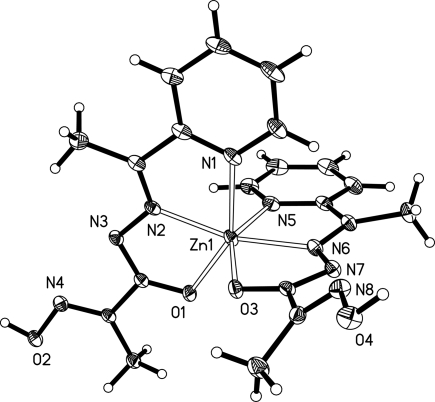
1 A view of compound 1, with displacement ellipsoids shown at the 40% probability level.

**Fig. 2.**
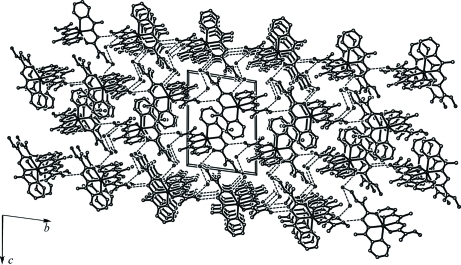
A packing diagram for 1 viewed in projection down the a axis. Hydrogen bonds are indicated by dashed lines; H atoms are omitted for clarity.

## supplementary crystallographic information

### Comment

As a part of our study of coordination compounds based on oxime-containing Schiff bases we would like to present the structure of the title compound 1, Fig. 1, which is based on polynucleative strand-type ligand 2-hydroxyimino-N'-[1-(2-pyridyl)ethylidene]propanohydrazone (Hpop) (Fig. 1). It has been shown previously that Hpop is able to form mono- and tetranuclear [2 x 2] grid-like assemblies with 3d-metal ions (Moroz et al., 2008a,b).

The title compound consists of neutral complex molecules and solvating water molecules. Zinc ion has a distorted tetragonal bipyramidal geometry. The coordination polyhedron is formed by two oxygen atoms from the amide groups and four nitrogen atoms belonging to two azomethine and two pyridine groups. The Zn—N and Zn—O bond lengths are comparable to previously reported zinc complexes with thiosemicarbasone and semicarbasone derivatives (Kasuga et al., (2003)), ligands with pyridine groups complexed to the metal ion (Petrusenko et al. (1997), Comba et al., (2002)) and the zinc-containing complex based on Hpop (Moroz et al., 2008b) (Table 1). The bite angles around the central atom deviate from an ideal square-planar configuration, that is a consequence of the formation of four almost flat five-membered chelate rings (Table 1). The ligands exist in complex molecule in singly charged form due to deprotonation of the amide group, C–N, C–O and N–N' bond distances are typical for deprotonated functions. In Hpop the oxime group is situated in anti- position to the amide group which was early shown in the structures of the free ligand and similiar compounds - amide derivatives of 2-hydroxyiminopropanoic acid (Onindo et al. (1995); Sliva et al. (1997a,b); Mokhir et al. (2002); Moroz et al., 2009a,b).

In the crystal packing the molecules of 1 form columns along a crystallographic direction due to hydrogen bonds and π-stacking interaction (Fig. 2). The columns are connected in 3D structure by a variety of hydrogen bonds where solvated water molecules act as donors and O and N atoms of the oxime group and O atom of the amide group of the ligand act as acceptors (Table 2).

### Experimental

Zinc(II) acetate (0.011 g, 0.05 mmol) in 5 ml H_2_O was added to 10 ml of hot methanol solution of Hpop (0.022 g, 0.1 mmol) and followed by 1 ml of alkali solution (0.1 M KOH). The mixture was left for slow evaporation at room temperature. After 5 days cubic yellowish crystals of 1 suitable for X-ray analysis were obtained.

### Refinement

The H_2_O hydrogen atoms were located from the difference Fourier map but constrained to ride on their parent atom, with U_iso_ = 1.5 U_eq_(parent atom). Other hydrogen atoms were positioned geometrically and were also constrained to ride on their parent atoms, with C—H = 0.95-0.98 Å, and U_iso_ = 1.2-1.5 U_eq_(parent atom). The highest peak is located 1.15 Å from atom H5O and the deepest hole is located 0.82 Å from atom Zn1.

### Crystal data

**Table:**

[Zn(C_10_H_11_N_4_O_2_)_2_]·2H_2_O	*Z* = 2
*M**_r_* = 539.86	*F*(000) = 560
Triclinic, *P*1	*D*_x_ = 1.580 Mg m^−^^3^
Hall symbol: -P 1	Mo *K*α radiation, λ = 0.71073 Å
*a* = 8.3241 (3) Å	Cell parameters from 4952 reflections
*b* = 10.6299 (4) Å	θ = 1.0–27.5°
*c* = 13.9006 (5) Å	µ = 1.14 mm^−^^1^
α = 94.184 (2)°	*T* = 100 K
β = 101.389 (2)°	Needle, yellow
γ = 108.052 (2)°	0.28 × 0.07 × 0.02 mm
*V* = 1134.48 (7) Å^3^	

### Data collection

**Table:**

Nonius KappaCCD diffractometer	5171 independent reflections
Radiation source: fine-focus sealed tube	4253 reflections with *I* > 2σ(*I*)
horizontally mounted graphite crystal	*R*_int_ = 0.048
Detector resolution: 9 pixels mm^-1^	θ_max_ = 27.5°, θ_min_ = 2.4°
φ scans and ω scans with κ offset	*h* = −10→10
Absorption correction: multi-scan (*SADABS*; Version 2008/1; Sheldrick, 2008)	*k* = −13→13
*T*_min_ = 0.743, *T*_max_ = 0.977	*l* = −18→18
21551 measured reflections	

### Refinement

**Table:**

Refinement on *F*^2^	Primary atom site location: structure-invariant direct methods
Least-squares matrix: full	Secondary atom site location: difference Fourier map
*R*[*F*^2^ > 2σ(*F*^2^)] = 0.036	Hydrogen site location: inferred from neighbouring sites
*wR*(*F*^2^) = 0.092	H-atom parameters constrained
*S* = 1.05	*w* = 1/[σ^2^(*F*_o_^2^) + (0.0417*P*)^2^ + 0.8841*P*] where *P* = (*F*_o_^2^ + 2*F*_c_^2^)/3
5171 reflections	(Δ/σ)_max_ < 0.001
320 parameters	Δρ_max_ = 0.95 e Å^−^^3^
0 restraints	Δρ_min_ = −0.39 e Å^−^^3^

### Special details

**Table:**

Geometry. All esds (except the esd in the dihedral angle between two l.s. planes) are estimated using the full covariance matrix. The cell esds are taken into account individually in the estimation of esds in distances, angles and torsion angles; correlations between esds in cell parameters are only used when they are defined by crystal symmetry. An approximate (isotropic) treatment of cell esds is used for estimating esds involving l.s. planes.
Refinement. Refinement of *F*^2^ against ALL reflections. The weighted *R*-factor wR and goodness of fit *S* are based on *F*^2^, conventional *R*-factors *R* are based on *F*, with *F* set to zero for negative *F*^2^. The threshold expression of *F*^2^ > σ(*F*^2^) is used only for calculating *R*-factors(gt) etc. and is not relevant to the choice of reflections for refinement. *R*-factors based on *F*^2^ are statistically about twice as large as those based on *F*, and *R*- factors based on ALL data will be even larger.

### Fractional atomic coordinates and isotropic or equivalent isotropic displacement parameters (Å^2^)

**Table:**

	*x*	*y*	*z*	*U*_iso_*/*U*_eq_	
Zn1	0.39746 (3)	0.27581 (3)	0.304853 (19)	0.01820 (9)	
O1	0.16169 (19)	0.20226 (15)	0.34643 (12)	0.0202 (3)	
O2	−0.32670 (19)	−0.16202 (17)	0.32111 (13)	0.0239 (4)	
H2O	−0.3576	−0.2526	0.3012	0.036*	
O3	0.30726 (19)	0.37780 (16)	0.18911 (12)	0.0204 (3)	
O4	0.4981 (2)	0.70910 (18)	0.00903 (13)	0.0290 (4)	
H4O	0.6065	0.7601	0.0013	0.044*	
O5	0.1803 (2)	0.1858 (2)	0.01793 (14)	0.0367 (4)	
H5P	0.2119	0.2596	0.0646	0.055*	
H5O	0.1264	0.1984	−0.0383	0.055*	
O6	0.0879 (2)	0.29498 (19)	−0.16109 (14)	0.0351 (4)	
H6O	0.0697	0.2177	−0.2017	0.053*	
H6P	0.2077	0.3384	−0.1471	0.053*	
N1	0.5988 (2)	0.2424 (2)	0.22445 (14)	0.0207 (4)	
N2	0.3039 (2)	0.07661 (19)	0.24489 (14)	0.0175 (4)	
N3	0.1450 (2)	0.00054 (19)	0.25994 (14)	0.0181 (4)	
N4	−0.1642 (2)	−0.11100 (19)	0.29900 (15)	0.0205 (4)	
N5	0.5549 (2)	0.2876 (2)	0.45404 (14)	0.0212 (4)	
N6	0.5524 (2)	0.4762 (2)	0.34417 (14)	0.0190 (4)	
N7	0.5460 (2)	0.55914 (19)	0.27198 (14)	0.0188 (4)	
N8	0.5314 (3)	0.6441 (2)	0.09127 (15)	0.0230 (4)	
C1	0.7541 (3)	0.3275 (3)	0.22043 (18)	0.0258 (5)	
H1	0.7857	0.4185	0.2482	0.031*	
C2	0.8707 (3)	0.2868 (3)	0.17679 (19)	0.0306 (6)	
H2	0.9811	0.3486	0.1767	0.037*	
C3	0.8235 (3)	0.1560 (3)	0.13393 (18)	0.0288 (6)	
H3	0.9005	0.1263	0.1035	0.035*	
C4	0.6620 (3)	0.0680 (3)	0.13569 (18)	0.0255 (5)	
H4	0.6257	−0.0224	0.1055	0.031*	
C5	0.5538 (3)	0.1148 (2)	0.18269 (16)	0.0203 (5)	
C6	0.3825 (3)	0.0232 (2)	0.19170 (16)	0.0190 (5)	
C7	0.3161 (3)	−0.1184 (3)	0.14258 (19)	0.0264 (5)	
H7A	0.3898	−0.1670	0.1744	0.040*	
H7B	0.3181	−0.1209	0.0722	0.040*	
H7C	0.1966	−0.1606	0.1488	0.040*	
C8	0.0873 (3)	0.0788 (2)	0.31405 (16)	0.0165 (4)	
C9	−0.0868 (3)	0.0123 (2)	0.33642 (16)	0.0174 (4)	
C10	−0.1580 (3)	0.0927 (2)	0.39855 (18)	0.0223 (5)	
H10A	−0.2852	0.0583	0.3795	0.034*	
H10B	−0.1168	0.1865	0.3883	0.034*	
H10C	−0.1187	0.0860	0.4686	0.034*	
C11	0.5453 (3)	0.1915 (3)	0.51063 (18)	0.0249 (5)	
H11	0.4600	0.1061	0.4865	0.030*	
C12	0.6559 (3)	0.2109 (3)	0.60431 (19)	0.0302 (6)	
H12	0.6500	0.1389	0.6417	0.036*	
C13	0.7726 (3)	0.3356 (3)	0.64108 (19)	0.0305 (6)	
H13	0.8473	0.3514	0.7051	0.037*	
C14	0.7817 (3)	0.4389 (3)	0.58458 (18)	0.0270 (5)	
H14	0.8602	0.5265	0.6099	0.032*	
C15	0.6724 (3)	0.4112 (2)	0.48927 (17)	0.0212 (5)	
C16	0.6797 (3)	0.5119 (2)	0.42118 (16)	0.0194 (5)	
C17	0.8289 (3)	0.6397 (2)	0.44150 (18)	0.0255 (5)	
H17A	0.8476	0.6708	0.3786	0.038*	
H17B	0.9338	0.6250	0.4774	0.038*	
H17C	0.8032	0.7074	0.4818	0.038*	
C18	0.4150 (3)	0.4947 (2)	0.19584 (17)	0.0183 (4)	
C19	0.3910 (3)	0.5686 (2)	0.10840 (17)	0.0199 (5)	
C20	0.2114 (3)	0.5440 (3)	0.04913 (19)	0.0290 (6)	
H20A	0.2124	0.6132	0.0061	0.043*	
H20B	0.1349	0.5468	0.0940	0.043*	
H20C	0.1688	0.4560	0.0083	0.043*	

### Atomic displacement parameters (Å^2^)

**Table:**

	*U*^11^	*U*^22^	*U*^33^	*U*^12^	*U*^13^	*U*^23^
Zn1	0.01308 (13)	0.01868 (15)	0.01989 (15)	0.00111 (10)	0.00272 (10)	0.00585 (10)
O1	0.0168 (8)	0.0165 (8)	0.0252 (9)	0.0013 (6)	0.0066 (6)	0.0030 (7)
O2	0.0147 (8)	0.0208 (9)	0.0335 (10)	−0.0003 (7)	0.0097 (7)	0.0043 (7)
O3	0.0172 (8)	0.0173 (8)	0.0217 (8)	0.0009 (6)	0.0003 (6)	0.0054 (7)
O4	0.0324 (9)	0.0346 (10)	0.0252 (9)	0.0134 (8)	0.0100 (7)	0.0171 (8)
O5	0.0357 (10)	0.0396 (12)	0.0305 (10)	0.0057 (9)	0.0089 (8)	0.0057 (9)
O6	0.0248 (9)	0.0351 (11)	0.0401 (11)	0.0065 (8)	0.0062 (8)	−0.0079 (9)
N1	0.0138 (9)	0.0276 (11)	0.0197 (10)	0.0043 (8)	0.0034 (7)	0.0100 (8)
N2	0.0132 (8)	0.0207 (10)	0.0195 (10)	0.0047 (7)	0.0051 (7)	0.0083 (8)
N3	0.0134 (9)	0.0175 (10)	0.0224 (10)	0.0016 (7)	0.0067 (7)	0.0050 (8)
N4	0.0154 (9)	0.0199 (10)	0.0255 (10)	0.0022 (8)	0.0084 (8)	0.0050 (8)
N5	0.0182 (9)	0.0266 (11)	0.0214 (10)	0.0080 (8)	0.0076 (8)	0.0088 (8)
N6	0.0158 (9)	0.0230 (10)	0.0161 (9)	0.0039 (8)	0.0025 (7)	0.0038 (8)
N7	0.0169 (9)	0.0198 (10)	0.0183 (9)	0.0038 (8)	0.0035 (7)	0.0065 (8)
N8	0.0282 (11)	0.0239 (11)	0.0213 (10)	0.0116 (9)	0.0080 (8)	0.0113 (8)
C1	0.0178 (11)	0.0333 (14)	0.0240 (12)	0.0037 (10)	0.0042 (9)	0.0119 (11)
C2	0.0159 (11)	0.0499 (18)	0.0265 (13)	0.0069 (11)	0.0083 (10)	0.0175 (12)
C3	0.0204 (12)	0.0486 (17)	0.0238 (13)	0.0153 (12)	0.0098 (10)	0.0134 (12)
C4	0.0224 (12)	0.0377 (15)	0.0207 (12)	0.0136 (11)	0.0072 (10)	0.0092 (11)
C5	0.0171 (11)	0.0318 (14)	0.0146 (11)	0.0095 (10)	0.0044 (9)	0.0112 (10)
C6	0.0156 (10)	0.0267 (13)	0.0153 (11)	0.0068 (9)	0.0037 (8)	0.0071 (9)
C7	0.0252 (12)	0.0289 (14)	0.0269 (13)	0.0087 (10)	0.0110 (10)	0.0032 (11)
C8	0.0141 (10)	0.0185 (11)	0.0163 (11)	0.0044 (9)	0.0023 (8)	0.0070 (9)
C9	0.0132 (10)	0.0196 (12)	0.0189 (11)	0.0039 (9)	0.0040 (8)	0.0060 (9)
C10	0.0161 (11)	0.0220 (12)	0.0270 (12)	0.0026 (9)	0.0072 (9)	0.0019 (10)
C11	0.0239 (12)	0.0290 (14)	0.0259 (13)	0.0099 (10)	0.0104 (10)	0.0115 (10)
C12	0.0324 (14)	0.0428 (16)	0.0264 (13)	0.0204 (12)	0.0137 (11)	0.0198 (12)
C13	0.0254 (13)	0.0471 (17)	0.0229 (13)	0.0151 (12)	0.0068 (10)	0.0128 (12)
C14	0.0197 (11)	0.0385 (15)	0.0204 (12)	0.0073 (10)	0.0022 (9)	0.0057 (11)
C15	0.0149 (10)	0.0300 (13)	0.0189 (11)	0.0074 (9)	0.0052 (9)	0.0035 (10)
C16	0.0154 (10)	0.0240 (12)	0.0164 (11)	0.0041 (9)	0.0028 (8)	0.0015 (9)
C17	0.0200 (11)	0.0252 (13)	0.0238 (12)	0.0005 (10)	−0.0001 (9)	0.0016 (10)
C18	0.0136 (10)	0.0202 (12)	0.0218 (11)	0.0053 (9)	0.0054 (9)	0.0054 (9)
C19	0.0207 (11)	0.0176 (11)	0.0205 (11)	0.0058 (9)	0.0034 (9)	0.0029 (9)
C20	0.0241 (12)	0.0321 (15)	0.0277 (13)	0.0083 (11)	−0.0011 (10)	0.0096 (11)

### Geometric parameters (Å, °)

**Table:**

Zn1—N2	2.061 (2)	C3—C4	1.385 (3)
Zn1—N6	2.085 (2)	C3—H3	0.9500
Zn1—O1	2.0880 (15)	C4—C5	1.396 (3)
Zn1—O3	2.1470 (15)	C4—H4	0.9500
Zn1—N5	2.1955 (19)	C5—C6	1.492 (3)
Zn1—N1	2.2877 (19)	C6—C7	1.491 (3)
O1—C8	1.268 (3)	C7—H7A	0.9800
O2—N4	1.397 (2)	C7—H7B	0.9800
O2—H2O	0.9213	C7—H7C	0.9800
O3—C18	1.272 (3)	C8—C9	1.508 (3)
O4—N8	1.404 (2)	C9—C10	1.495 (3)
O4—H4O	0.9287	C10—H10A	0.9800
O5—H5P	0.9140	C10—H10B	0.9800
O5—H5O	0.8626	C10—H10C	0.9800
O6—H6O	0.9154	C11—C12	1.398 (4)
O6—H6P	0.9335	C11—H11	0.9500
N1—C5	1.342 (3)	C12—C13	1.367 (4)
N1—C1	1.343 (3)	C12—H12	0.9500
N2—C6	1.287 (3)	C13—C14	1.388 (4)
N2—N3	1.385 (2)	C13—H13	0.9500
N3—C8	1.337 (3)	C14—C15	1.404 (3)
N4—C9	1.284 (3)	C14—H14	0.9500
N5—C11	1.327 (3)	C15—C16	1.475 (3)
N5—C15	1.357 (3)	C16—C17	1.492 (3)
N6—C16	1.287 (3)	C17—H17A	0.9800
N6—N7	1.387 (3)	C17—H17B	0.9800
N7—C18	1.325 (3)	C17—H17C	0.9800
N8—C19	1.274 (3)	C18—C19	1.508 (3)
C1—C2	1.398 (4)	C19—C20	1.491 (3)
C1—H1	0.9500	C20—H20A	0.9800
C2—C3	1.375 (4)	C20—H20B	0.9800
C2—H2	0.9500	C20—H20C	0.9800
			
N2—Zn1—N6	162.52 (7)	C6—C7—H7A	109.5
N2—Zn1—O1	76.10 (7)	C6—C7—H7B	109.5
N6—Zn1—O1	121.37 (7)	H7A—C7—H7B	109.5
N2—Zn1—O3	105.12 (7)	C6—C7—H7C	109.5
N6—Zn1—O3	74.17 (6)	H7A—C7—H7C	109.5
O1—Zn1—O3	96.43 (6)	H7B—C7—H7C	109.5
N2—Zn1—N5	106.20 (7)	O1—C8—N3	127.01 (19)
N6—Zn1—N5	75.07 (7)	O1—C8—C9	117.25 (19)
O1—Zn1—N5	93.88 (7)	N3—C8—C9	115.72 (19)
O3—Zn1—N5	148.54 (7)	N4—C9—C10	124.75 (19)
N2—Zn1—N1	73.97 (7)	N4—C9—C8	116.43 (19)
N6—Zn1—N1	88.55 (7)	C10—C9—C8	118.82 (19)
O1—Zn1—N1	150.07 (7)	C9—C10—H10A	109.5
O3—Zn1—N1	90.90 (6)	C9—C10—H10B	109.5
N5—Zn1—N1	94.81 (7)	H10A—C10—H10B	109.5
C8—O1—Zn1	111.22 (13)	C9—C10—H10C	109.5
N4—O2—H2O	103.0	H10A—C10—H10C	109.5
C18—O3—Zn1	110.35 (13)	H10B—C10—H10C	109.5
N8—O4—H4O	105.6	N5—C11—C12	122.6 (2)
H5P—O5—H5O	111.3	N5—C11—H11	118.7
H6O—O6—H6P	104.7	C12—C11—H11	118.7
C5—N1—C1	118.2 (2)	C13—C12—C11	118.7 (2)
C5—N1—Zn1	112.02 (14)	C13—C12—H12	120.7
C1—N1—Zn1	129.54 (18)	C11—C12—H12	120.7
C6—N2—N3	119.76 (19)	C12—C13—C14	119.8 (2)
C6—N2—Zn1	123.02 (15)	C12—C13—H13	120.1
N3—N2—Zn1	117.15 (14)	C14—C13—H13	120.1
C8—N3—N2	108.51 (18)	C13—C14—C15	118.6 (2)
C9—N4—O2	112.04 (18)	C13—C14—H14	120.7
C11—N5—C15	119.1 (2)	C15—C14—H14	120.7
C11—N5—Zn1	127.97 (17)	N5—C15—C14	121.1 (2)
C15—N5—Zn1	112.92 (14)	N5—C15—C16	116.1 (2)
C16—N6—N7	120.20 (19)	C14—C15—C16	122.7 (2)
C16—N6—Zn1	119.99 (16)	N6—C16—C15	114.0 (2)
N7—N6—Zn1	117.29 (14)	N6—C16—C17	125.2 (2)
C18—N7—N6	108.84 (18)	C15—C16—C17	120.8 (2)
C19—N8—O4	111.39 (19)	C16—C17—H17A	109.5
N1—C1—C2	122.3 (3)	C16—C17—H17B	109.5
N1—C1—H1	118.9	H17A—C17—H17B	109.5
C2—C1—H1	118.9	C16—C17—H17C	109.5
C3—C2—C1	119.1 (2)	H17A—C17—H17C	109.5
C3—C2—H2	120.5	H17B—C17—H17C	109.5
C1—C2—H2	120.5	O3—C18—N7	126.8 (2)
C2—C3—C4	119.1 (2)	O3—C18—C19	116.84 (19)
C2—C3—H3	120.4	N7—C18—C19	116.34 (19)
C4—C3—H3	120.4	N8—C19—C20	126.5 (2)
C3—C4—C5	118.7 (3)	N8—C19—C18	114.9 (2)
C3—C4—H4	120.6	C20—C19—C18	118.58 (19)
C5—C4—H4	120.6	C19—C20—H20A	109.5
N1—C5—C4	122.5 (2)	C19—C20—H20B	109.5
N1—C5—C6	116.25 (19)	H20A—C20—H20B	109.5
C4—C5—C6	121.2 (2)	C19—C20—H20C	109.5
N2—C6—C7	125.1 (2)	H20A—C20—H20C	109.5
N2—C6—C5	114.6 (2)	H20B—C20—H20C	109.5
C7—C6—C5	120.3 (2)		

### Hydrogen-bond geometry (Å, °)

**Table:**

*D*—H···*A*	*D*—H	H···*A*	*D*···*A*	*D*—H···*A*
O2—H2O···N7^i^	0.92	1.89	2.801 (3)	170
O4—H4O···O5^ii^	0.93	1.77	2.675 (3)	164
O5—H5P···O3	0.91	1.93	2.811 (3)	161
O5—H5O···O6	0.86	2.08	2.889 (3)	157
O6—H6O···N4^iii^	0.92	2.12	2.934 (3)	148
O6—H6P···N8^ii^	0.93	2.10	2.971 (3)	154

Symmetry codes: (i) *x*−1, *y*−1, *z*; (ii) −*x*+1, −*y*+1, −*z*; (iii) −*x*, −*y*, −*z*.
